# Add-on Sodium Benzoate and *N*-Acetylcysteine in Patients With Early Schizophrenia Spectrum Disorder: A Multicenter, Double-Blind, Randomized Placebo-Controlled Feasibility Trial

**DOI:** 10.1093/schizbullopen/sgae004

**Published:** 2024-02-09

**Authors:** Muhammad Omair Husain, Imran Bashir Chaudhry, Ameer B Khoso, Muhammad Ishrat Husain, Moin Ahmed Ansari, Nasir Mehmood, Haider A Naqvi, Asad Tamizuddin Nizami, Uroosa Talib, Aatir H Rajput, Paul Bassett, George Foussias, Bill Deakin, Nusrat Husain

**Affiliations:** Campbell Family Mental Health Research Institute, Centre for Addiction and Mental Health, Toronto, ON, Canada; Department of Psychiatry, Temerty Faculty of Medicine, University of Toronto, Toronto, ON, Canada; Division of Psychology and Mental Health, University of Manchester, Manchester, UK; Department of Psychiatry, Ziauddin University, Karachi, Pakistan; Pakistan Institute of Living and Learning, Karachi, Pakistan; Pakistan Institute of Living and Learning, Karachi, Pakistan; Division of Population Health, Health Services Research and Primary Care, School of Health Sciences, Faculty of Biology, Medicine and Health, University of Manchester, UK; Campbell Family Mental Health Research Institute, Centre for Addiction and Mental Health, Toronto, ON, Canada; Department of Psychiatry, Temerty Faculty of Medicine, University of Toronto, Toronto, ON, Canada; Department of Psychiatry, Liaquat University of Medical and Health Sciences, Hyderabad, Pakistan; Karwan e Hayat, Institute for Mental Health Care, Karachi, Pakistan; Department of Psychiatry, Dow University Health Sciences, Karachi, Pakistan; Institute of Psychiatry, WHO Collaborating Centre for Mental Health Research and Training, Rawalpindi Medical University, Rawalpindi, Pakistan; Karwan e Hayat, Institute for Mental Health Care, Karachi, Pakistan; Department of Psychiatry, Liaquat University of Medical and Health Sciences, Hyderabad, Pakistan; StatsConsultancy, Amersham, UK; Campbell Family Mental Health Research Institute, Centre for Addiction and Mental Health, Toronto, ON, Canada; Department of Psychiatry, Temerty Faculty of Medicine, University of Toronto, Toronto, ON, Canada; Division of Psychology and Mental Health, University of Manchester, Manchester, UK; Division of Psychology and Mental Health, University of Manchester, Manchester, UK; Mersey Care NHS Foundation Trust, Prescott, UK; Institute of Population and Mental Health, University of Liverpool, Liverpool, UK

**Keywords:** early schizophrenia, inflammation, LMIC, *N*-acetylcysteine, Sodium Benzoate, Pakistan

## Abstract

**Background and Hypothesis:**

Oxidative stress pathways may play a role in schizophrenia through direct neuropathic actions, microglial activation, inflammation, and by interfering with NMDA neurotransmission. *N*-acetylcysteine (NAC) has been shown to improve negative symptoms of schizophrenia, however, results from trials of other compounds targeting NMDA neurotransmission have been mixed. This may reflect poor target engagement but also that risk mechanisms act in parallel. Sodium Benzoate (NaB) could have an additive with NAC to act on several pathophysiological mechanisms implicated in schizophrenia.

**Study Design:**

A multicenter, 12 weeks, 2 × 2 factorial design, randomized double-blind placebo-controlled feasibility trial of NaB and NAC added to standard treatment in 68 adults with early schizophrenia. Primary feasibility outcomes included recruitment, retention, and completion of assessments as well as acceptability of the study interventions. Psychosis symptoms, functioning, and cognitive assessments were also assessed.

**Study Results:**

We recruited our desired sample (*n* = 68) and retained 78% (*n* = 53) at 12 weeks, supporting the feasibility of recruitment and retention. There were no difficulties in completing clinical outcome schedules. Medications were well tolerated with no dropouts due to side effects. This study was not powered to detect clinical effect and as expected no main effects were found on the majority of clinical outcomes.

**Conclusions:**

We demonstrated feasibility of conducting a clinical trial of NaB and NAC. Given the preliminary nature of this study, we cannot draw firm conclusions about the clinical efficacy of either agent, and a large-scale trial is needed to examine if significant differences between treatment groups emerge.

**Trial Registration:**

ClinicalTrials.gov: NCT03510741.

**Key Points**

- The primary focus of this study was to establish the feasibility of a multicenter randomized controlled trial, employing a factorial design, of Sodium Benzoate and/or *N*-acetylcysteine added to standard treatment in patients with early schizophrenia spectrum disorder.
- Feasibility was informed by recruitment and retention rates, as well as tolerability of trial drugs.
- To our knowledge this is the first study investigating the combination of these compounds.
- Given the preliminary nature of this study, we cannot draw firm conclusions about the clinical efficacy of either agent and a large-scale trial with longer duration of follow-up may be needed to examine if significant differences between treatment groups emerge.

## Background

Schizophrenia is the most common psychotic disorder worldwide. Although outcomes vary, the disorder usually has a severe and enduring course, associated with poor long-term outcomes, especially in terms of functioning.^1,2^ Although, both first and second-generation antipsychotics, are efficacious in treating positive symptoms, their effects on negative symptoms and cognitive impairment are minimal at best.^3^ Symptoms within these 2 domains have a significant impact on functioning and quality of life.^4^ In addition to the dopaminergic hypothesis of schizophrenia, N-methyl-D-aspartate (NMDA) receptor hypofunctioning is thought to be implicated in the pathogenesis of the disease.^5–8^ The schizophrenia-like state induced by NMDA receptor antagonists, ketamine, and phencyclidine, led to suggestions of the glutaminergic system playing a vital pathophysiological role. These drugs produce both positive and negative symptoms, alongside cognitive dysfunction.^7^ The NMDA receptor has significant roles in neurocognition and neurotoxicity.^9^ There have been encouraging reports of treatments targeting the NMDA receptor.^10^ NMDA-enhancing agents have been reported to have positive findings, albeit with limited efficacy.^10–14^ A meta-analysis of 26 studies, found that NMDA receptor enhancing treatments have modest effect sizes across both positive and negative symptoms.^15^ An alternative means of modulating the function of NMDA receptors is by inhibiting d-amino acid oxidase (DAAO), thereby increasing synaptic levels of d-amino acids.^16,17^

DAAO is found to be elevated in postmortem studies of adults with schizophrenia.^18,19^ Sodium Benzoate (NaB) is a DAAO inhibitor, and its salts are recognized as being safe by the United States Food and Drug Administration (FDA). NaB can potentially modulate the function of NMDA receptors by inhibiting DAAO, thereby increasing synaptic levels of d-amino acids.^16,17^ A previous study found NaB to improve a variety of symptoms and cognitive function, in adults with schizophrenia, when added to standard antipsychotic treatment.^9^ A more recent randomized controlled trial (RCT) aimed to examine the effectiveness and safety of NaB, for adults with schizophrenia who had partial response to clozapine.^20^ NaB demonstrated improvement on the Scale for the Assessment of Negative Symptoms (SANS), when compared to placebo.^20^ It has been reported that NaB also exerts anti-inflammatory effects, reducing microglial and astroglial inflammatory responses through the attenuation of inducible nitric oxide sythnase (iNOS^21^). NaB reduces in vivo cholesterol levels in mice and depletion of intermediates of the mevalonate pathway is also thought to contribute to an anti-inflammatory effect.^21^ In this regard it resembles the action of statins which have shown benefit on negative symptoms in a previous study from our group,^22^ and in reducing gray matter loss in multiple sclerosis.^23^ NaB’s potential for therapeutic use in schizophrenia may be exerted by a combination of its anti-inflammatory and NMDA receptor activating properties.

NAC (*N*-acetylcysteine), the precursor of the body’s main antioxidant, glutathione (GSH), is of therapeutic interest for a number of neuropsychiatric disorders. GSH neutralizes reactive oxygen species which, like peroxytnitites, can be neurotoxic but may also have a physiological role in neurotransmitter release.^24^ There is evidence that glutathione is decreased in the prefrontal cortex and cerebrospinal fluid of drug-naive patients with schizophrenia.^25^ NAC also acts on the glutamate/cysteine antiporter modulating levels of extracellular glutamate.^26^ In a 6-month placebo-controlled double-blind RCT of 140 adults with chronic schizophrenia, 2 g of NAC daily added to standard treatment, was associated with significant reduction on Positive and Negative Syndrome Scale (PANSS) scores, improved Clinical Global Impression (CGI), and functioning scales.^27^ A significant proportion of the participants in this study were considered treatment-resistant, with more than 45% of participants treated with clozapine with an illness duration of 12 years.^27^ Another study found NAC to be both effective and safe, as an add on treatment to risperidone for negative symptoms of schizophrenia.^28^

Finding effective novel treatments, particularly for negative symptoms of schizophrenia is vital, due to the impact they have on functional outcomes.^1^ NAC, and to a lesser degree, NaB, have been identified as agents that can alleviate positive and negative symptoms of schizophrenia. It is important that pharmacotherapeutic agents for neuropsychiatric disorders have permeability across the blood brain barrier, and both NAC and NaB have this property.^29,30^ We hypothesize that the glutaminergic effects of both compounds may synergize and the additional antioxidative properties of NAC may have an additive effect in improving psychopathology in individuals with early schizophrenia. The best predictor of future quality of life and occupational functioning is the initial severity of negative symptoms, along with the duration of untreated psychosis.^1,31^ In this context, we assessed the feasibility of a 12 weeks, 2 × 2 factorial design RCT of NaB and NAC (alone and in combination) added to standard treatment in adults with early schizophrenia spectrum disorder by evaluating recruitment, adherence, retention, attrition, tolerability of drugs, and completeness of the outcome measures at each data collection point.

## Aims and Objectives

1. Primary aims: To establish the feasibility of conducting a factorial design randomized control trial of NaB and NAC (alone and in combination) added to standard treatment in adults with early schizophrenia spectrum disorder by evaluating recruitment, adherence, retention, attrition, tolerability of drugs, and completeness of the outcome measures at each data collection point.
2. Secondary aims: To establish the preliminary clinical efficacy of NaB and NAC (alone and in combination) added to standard treatment in adults with early schizophrenia spectrum disorder in improving global symptoms of psychosis.
3. Additional aims: To establish the preliminary clinical efficacy of NaB and NAC (alone and in combination) added to standard treatment in adults with early schizophrenia spectrum disorder in improving positive symptoms, negative symptoms, overall symptom severity, social functioning, occupational functioning, and cognitive functioning.

## Methods

### Study Design

This is a 12-week double-blind clinical trial in which 68 adults with early schizophrenia spectrum disorder were randomized to: NaB plus placebo, NAC plus placebo, NaB plus NAC, or placebo plus placebo, added to standard treatment according to a 2 × 2 factorial design. A factorial design maximizes the number exposed to each drug, alone or in combination, vs those not exposed (main effects) while detecting benefits of combined treatment (interaction). The trial was registered on Clinicaltrial.gov (NCT03510741). Ethical approval was obtained from the National Bioethics Committee Pakistan (Approval Number: 4-87/NBC-282-Y2-Extension/19/1574). Local institutional approvals were obtained from all recruitment sites. Participants were recruited from major psychiatric units across major cities in Pakistan. Clinical staff from the participants’ own mental health teams continued to provide care during the course of the study. There was no withdrawal of existing interventions or treatments as part of this study. The participants were assessed with clinical scales, side-effect checklists, and neuropsychological assessments at baseline, 2, 4, 8, and 12 weeks. We aimed to recruit at least 16 participants per group to ensure that, even after loss to follow-up we would have at least 12 participants per group for analysis. The sample size was informed by published literature, which suggests a total of 24–50 participants for feasibility trials.^32,33^ Randomization was carried out by an independent statistician.

### Participants

The main inclusion criteria were male and female adults aged 18–35 years, with a diagnosis of schizophrenia confirmed by the Structured Clinical Interview for DSM-5 (SCID-5) criteria for schizophreniform disorder, schizophrenia, or schizoaffective disorder and within 5 years of onset of psychotic symptoms, as assessed by the research team. Participants were required to be receiving mental health care at one of the participating sites, and have been on a stable dose of antipsychotic medication for the preceding 4 weeks. We did not stipulate a minimum PANSS score to establish eligibility of participants. Participants were required to demonstrate the capacity to provide informed consent as assessed by their own clinician, be able to complete the required evaluations, and take oral medication. Participants of child-bearing age were required to use effective contraceptive precautions (either the use of barrier methods or the oral contraceptive pill) and a negative pregnancy test was required in order to meet inclusion criteria.

The main exclusion criteria were a prior history of intolerance or serious side effects to NaB or NAC, concomitant use of ascorbic acid, DSM-5 substance use disorder (except nicotine or caffeine) within the last 3 months, DSM-5 moderate to severe intellectual disability, comorbid unstable physical health, pregnancy, or breast feeding.

### Interventions

A pharmaceutical manufacturer in Pakistan, which holds ISO certification, manufactured the placebo and created matching NaB and NAC by over-encapsulation. All participants took 2 capsules twice a day: (1) active NAC or identical NAC placebo and (2) active NaB or identical NaB placebo. The pharmacy departments at each site dispensed the study medication. NaB was administered at 1000 mg daily and NAC 1000 mg twice daily dosing as informed by prior clinical trials of these compounds.^9,34^ Standard care of schizophrenia in Pakistan usually involves psychiatric follow-up and antipsychotic medication. Psychosocial interventions for schizophrenia are not routinely available in this setting. Treatment history of each participant was recorded at each visit. Changes to the participants standard treatment during the study period were permissible under the discretion of the participants’ responsible clinician. Responsible clinicians were encouraged to maintain stable standard care throughout the trial. Any changes in medication were documented by the research staff.

### Outcomes

#### Feasibility Outcomes

1. Recruitment rates, adherence (adherence with >80% medication as determined by pill counts^35^), retention, and randomization (proportion consented who are then randomized).
2. Acceptability of the intervention (tolerance, reported side effects, and attrition rates).
3. Completeness of assessment tools and the assessment schedule by participants.

#### Diagnostic, Clinical and Functional Assessments

1. SCID (at baseline only).^36,37^
2. PANSS.^38^ This scale has been used in previous and current trials in Pakistan by our group.^22,39–41^
3. CGI Scale—Severity.^42^ This scale was used in previous and current trials in Pakistan by our group.^22,39,40^
4. Social and Occupational Functioning Assessment Scale (SOFAS).^43^ This scale is being used in a current trial in Pakistan by our group.^44^

#### Cognitive Assessments

1. The Stroop Test^45^ is a test of divided attention and processing speed. The test has 2 conditions: “name the word” (T1, low processing load), and “name the color of the word” (T2, high processing load as the color of the ink may be different to the color depicted by the word itself). Scores represent time taken in seconds to complete the tasks.
2. The Oral Fluency test (Categories) involves executive function and linguistic skills. The participant names as many exemplars [a word or term] as they can think of pertaining to designated categories of item. There are 3 categories per test and 1 min is allowed per category. Scores represent the mean number of category exemplars per minute.
3. Coughlan Learning Task (verbal)^46^—After reading a list of words, the examiner asks the participant to repeat as many of the words they can remember as possible. This is repeated 5 times. A new list of words is then given. The participant is asked to repeat as many of the new words as possible. Once this has been done the participant is asked to recall as many of the words from the first list as they can remember. Scores represent total number of words recalled across trials.
4. Coughlan Learning Task (visual)^46^: Participants are shown a pattern design for 10 s before being asked to try and draw the same pattern from memory. This is repeated 5 times. Thereafter, they are shown a new pattern and asked to draw this as accurately as possible. Once completed, they are asked to draw the pattern that they were first shown. The score is based on the participant’s drawings and the total number of individual lines correctly reproduced across trials.

### Randomization and Masking

All participants were allocated to 1 of the 4 study arms based on a list prepared by an independent trial statistician. Only the trial pharmacist had access to treatment allocation for emergency unblinding authorized by the principal investigator or his deputy; unblinding did not prove necessary. The central trial pharmacist prepared bi-weekly packages of treatment bearing the participant name and ID number and sent it to the site pharmacy. Thus, the site pharmacy were blinded to treatment allocation.

### Procedures

Local clinical teams directed potential participants to the study research assistants (RAs) who screened for eligibility. A patient information leaflet with details of the study was given to potential participants and a meeting was arranged with the research team to explain the study in detail. At least 24 h were given prior to obtaining consent to participate in the study. Participants were informed that they were free to withdraw from the study at any time for any reason and that this would not impact their routine clinical care. Written informed consent was obtained from individuals willing to take part in the study. Clinical teams continued to provide usual care throughout the duration of the trial. Changes in medication were permissible, though stability was encouraged. RAs completed assessments for efficacy, safety, and tolerability at 2, 4, 8, and 12 weeks. We conducted pill counts at each follow-up assessment to assess treatment adherence. Inter-rater reliability (IRR) was assessed for PANSS assessments (*n* = 11) with an IRR score of 0.845. The trial was monitored by an independent Trial Steering Committee (TSC) that included a senior physician and a service user. The TSC also had the responsibility for data monitoring to oversee any potential harm to the participants from taking part in the trial.

### Statistical Analysis Plan

Given the feasibility nature of this trial, we did not power the study to show a statistically significant effect of NaB and NAC. We planned a descriptive analysis for demographic and clinical variables. Continuous variables were summarized by mean, and standard deviation, and/or medians and interquartile ranges as appropriate. Categorical variables were summarized by frequencies and percentages. The data were entered and analyzed using Stata (version 15.1).

The outcome variables were measured at 2, 4, 8, and 12 weeks. Due to the repeated measurements from each patient over time, the analyses were performed using mixed (multilevel) regression models. The baseline values of each corresponding outcome were included as a covariate in the analyses. The interaction between time and treatment was included in the model to allow for the effects at each timepoint to be quantified separately. The 4 groups were compared in a 1-way comparison at each time point. To take advantage of the 2 × 2 design, the main effects of a NaB (exposed vs nonexposed) factor and a NAC (exposed vs nonexposed) factor were also analyzed. No interaction analysis was planned due to the lack of power in this small feasibility study. Linear mixed models were used for the continuous outcomes, whilst a mixed ordinal logistic regression model was used for the ordinal outcome variables.

## Results

**Fig. 1.**
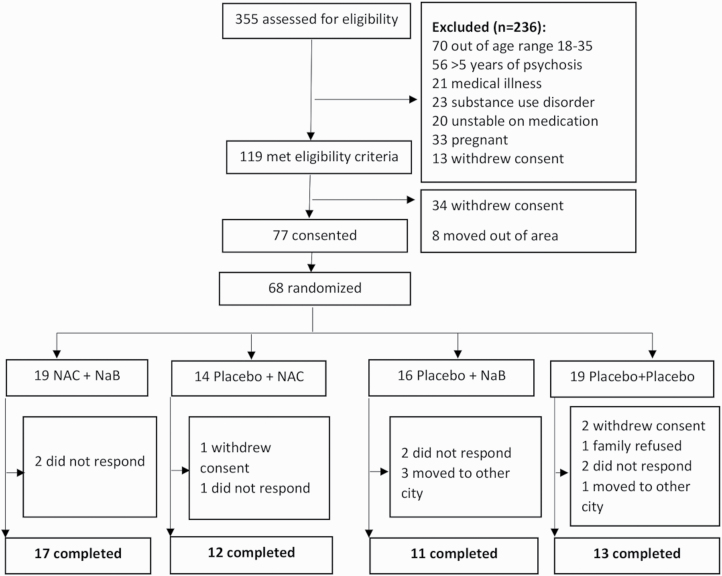
Trial profile.

A total of 355 patients were approached and assessed for eligibility between January 2019 and December 2020 (the Covid-19 pandemic significantly affected the recruitment process which resulted in delays in completing the study) of whom 119 met the eligibility criteria and were invited for participation, 77 consented to participate and 68 (88%) of those were randomly assigned to the 4 different treatment groups (figure 1, table 1). Of these participants, at the end of the trial 12-week visit, 15 (22%) had dropped out of the study. Just over 3-quarters (78%) remained in the study throughout. Of the 15 who dropped out of the study, 7 (46%) were no longer contactable, 4 (27%) relocated to another city, and 4 (27%) withdrew consent to participate. The demographic characteristics of the sample are described in table 1. The mean age between groups was comparable. There was a preponderance of females in 1 group, and majority of participants were married. Participants had between 5 and 7 years of education. The majority of participants were prescribed atypical antipsychotic medication (*n* = 41; 60.3%) and none were receiving psychological interventions. Of the individuals on atypical antipsychotics 6 were also prescribed low-dose clozapine (25 mg daily) in combination with Olanzapine. There were 8 participants who were not taking any antipsychotic medication, for reasons that have not been reported. The majority of participants were diagnosed with schizophrenia (*n* = 48; 70.6%) and there were no statistically significant differences between the groups with regards to duration of illness, the number of prior psychiatric hospitalizations, or of chlorpromazine equivalent doses of antipsychotic prescribed.^47^

**Table 1.** Demographic Characteristics

	NAC + NaB (*N* = 19)	Placebo (*N* = 19)	Placebo + NAC (*N* = 14)	Placebo + NaB (*N* = 16)	Total (*N* = 68)
Median [IQR]					
Age (in years)	27 [21–33]	28 [24–32]	30 [23–34]	27 [24–32]	28 [24–33]
Year of education	6 [5–10]	5 [0–8]	6.5 [3.7–12]	7.5 [0–9.5]	6.5 [0–10]
No of family members	6 [5–8]	8 [5–9]	6.5 [4.75–8.25]	7 [5.25–10]	6 [5–8]
Household income US dollars/month	35 [53–88]	53 [53–105]	87 [40–127]	70 [70–137]	53 [53–105]
Age at the time of illness (in years)	24 [18–31]	25 [21–29]	27 [19–30]	24 [21–27]	25 [20–29]
Duration of mental illness (in months)	36 [24–36]	36 [24–36]	36 [24–48]	36 [24–48]	36 [24–47]
Prior psychiatric hospitalization	1 [0–2]	0 [0–1]	0 [0–1]	0 [0–1.75]	0 [0–1]
*n* (%)					
Sex					
Male	4 (21.1%)	10 (52.6%)	7 (50.0%)	10 (62.5%)	31 (45.6%)
Female	15 (78.9%)	9 (47.4%)	7 (50.0%)	6 (37.5%)	37 (54.4%)
Marital status					
Single	8 (42.1%)	9 (47.4%)	7 (50.0%)	7 (43.8%)	31 (45.6%)
Married	11 (57.9%)	10 (52.6%)	6 (42.9%)	9 (56.3%)	36 (52.9%)
Separation/Divorced	0 (0.0%)	0 (0.0%)	1 (7.1%)	0 (0.0%)	1 (1.5%)
Family system					
Single	13 (68.4%)	10 (52.6%)	7 (50.0%)	10 (62.5%)	40 (58.8%)
Joint	6 (31.6%)	9 (47.4%)	7 (50.0%)	6 (37.5%)	28 (41.2%)
Diagnosis					
Schizophrenia	13 (68.4%)	12 (63.2%)	12 (85.7%)	11 (68.8%)	48 (70.6%)
Schizoaffective	03 (15.8%)	07 (36.8%)	02 (14.3%)	04 (25.0%)	16 (23.5%)
Schizophreniform	03 (15.8%)	0 (0.0%)	0 (0.0%)	01 (6.3%)	04 (5.9%)
Medication					
Typical antipsychotics only	1 (5.3%)	1 (5.3%)	0 (0.0%)	2 (12.5%)	4 (5.9%)
Atypical antipsychotics only	12 (63.2%)	11 (57.9%)	11 (78.6%)	7 (43.8%)	41 (60.3%)
Clozapine + atypical antipsychotic (Olanzapine)^a^	4 (21.1%)	01 (5.3%)	01 (7.1%)	0 (0.0%)	6 (8.8%)
Combination of atypical and typical antipsychotics	5 (26.3%)	05 (26.3%)	01 (7.1%)	04 (25.0%)	15 (21%)
No antipsychotics	01 (5.3%)	02 (10.5%)	2 (14.3%)	3 (18.8%)	08 (11.8%)
Number of participants with antipsychotic change over the 12-week study					
	2 (10.5%)	3 (15.8%)	1 (7.1%)	3 (18.8%)	9 (13.2%)
Mean ± *SD*					
Chlorpromazine equivalent doses of antipsychotics	254 ± 167	280 ± 172	161 ± 149	205 ± 186	231 ± 171

Data are presented Median [IQR], *N* (%) or Mean ± *SD*.

^a^Subgroup of individuals prescribed Atypical antipsychotics.

Medication adherence was calculated for the 53 of the 68 participants who remained in the study and was defined as >80% of capsules taken. This was calculated for each participant based on number of capsules dispensed and number returned, which was totaled at the end of the trial. Medications were dispensed at the same intervals as assessments, ie, baseline, 2, 4, 8, and 12 weeks. All participants who completed the trial took more than 80% of dispensed medication based on pill counts.

**Fig. 2.**
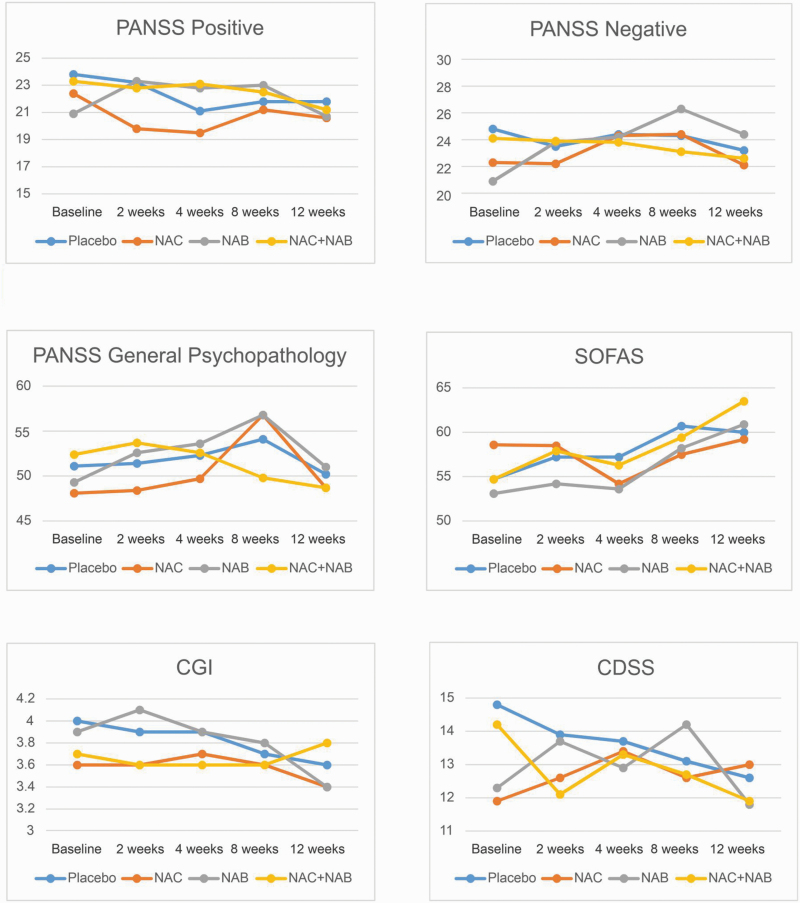
Graphical representation of group-based effects on PANSS scores, SOFAS, CGI, and CDSS.

Treatment efficacy endpoints and summary statistics for each of the 4 treatment conditions are shown in table 2 and figure 2. Side-effect ratings were also compared across the 4 individual treatment groups at all follow-ups time point. There were no significant differences across the groups and the most common side effects reported were headaches, irritability, and agitation (supplementary table 1). There largely was no statistically significant differences between the NaB and no NaB groups, with the exception of PANSS positive, where scores at 4 weeks were significantly higher in the NaB group than in the no NaB group (table 3). There was a mean difference of 2.5 units between groups. The difference between groups for this outcome was not significant at the other timepoints. There were no differences in cognitive measures (supplementary table 2).

**Table 2.** Interaction of NaB and NAC on PANSS Scores, CGI, SOFAS, and CDSS

Outcome	Time point	Placebo [*n* = 19]		NAC only [*n* = 14]		NaB only [*n* = 16]		NAC + NaB [*n* = 19]			*P-*value
		*n*	Mean ± *SD*	*n*	Mean ± *SD*	*n*	Mean ± *SD*	*n*	Mean ± *SD*	*F*	
PANSS positive	Baseline	19	23.8 ± 3.9	14	22.4 ± 5.3	16	20.9 ± 4.3	19	23.3 ± 4.4		
	2 weeks	18	23.2 ± 3.3	13	19.8 ± 4.7	12	23.3 ± 1.8	19	22.8 ± 6.2	1.799	.158
	4 weeks	18	21.1 ± 2.4	12	19.5 ± 4.6	11	22.8 ± 3.1	19	23.1 ± 4.6	2.636	.059
	8 weeks	14	21.8 ± 3.6	12	21.2 ± 5.5	11	23.0 ± 2.6	17	22.5 ± 4.9	0.417	.741
	12 weeks	13	21.8 ± 2.2	12	20.6 ± 5.3	11	20.7 ± 4.5	17	21.2 ± 4.1	.214	.887
PANSS negative	Baseline	19	24.8 ± 4.5	14	22.3 ± 5.1	16	20.9 ± 5.7	19	24.1 ± 4.7		
	2 weeks	18	23.5 ± 4.6	13	22.2 ± 4.6	12	23.8 ± 4.6	19	23.9 ± 3.8	0.398	.755
	4 weeks	18	24.4 ± 4.5	12	24.3 ± 4.5	11	24.2 ± 3.5	19	23.8 ± 3.2	0.069	.976
	8 weeks	14	24.3 ± 5.0	12	24.4 ± 2.7	11	26.3 ± 4.8	17	23.1 ± 2.8	1.469	.234
	12 weeks	13	23.2 ± 3.7	12	22.1 ± 5.3	11	24.4 ± 4.6	17	22.6 ± 2.3	0.666	.577
PANSS general	Baseline	19	51.1 ± 6.7	14	48.1 ± 5.2	16	49.3 ± 8.2	19	52.4 ± 9.5		
	2 weeks	18	51.4 ± 9.9	13	48.4 ± 8.8	12	52.6 ± 5.9	19	53.7 ± 8.4	1.057	.374
	4 weeks	18	52.3 ± 8.1	12	49.7 ± 7.6	11	53.6 ± 7.0	19	52.6 ± 8.6	0.539	.657
	8 weeks	14	54.1 ± 6.7	12	56.8 ± 7.8	11	56.8 ± 7.7	17	49.8 ± 6.6	2.281	.091
	12 weeks	13	50.2 ± 7.9	12	48.7 ± 13.4	11	51.0 ± 8.7	17	48.7 ± 6.4	0.200	.896
PANSS total	Baseline	19	99.6 ± 11.6	14	92.8 ± 10.3	16	91.1 ± 15.3	19	99.8 ± 17.2		
	2 weeks	18	98.1 ± 16.3	13	90.5 ± 15.9	12	99.8 ± 15.9	19	100.5 ± 16.3	1.279	.290
	4 weeks	18	97.8 ± 12.4	12	93.5 ± 11.2	11	100.6 ± 10.6	19	99.5 ± 14.9	0.744	.530
	8 weeks	14	100.1 ± 11.9	12	98.3 ± 14.0	11	106.1 ± 10.2	17	95.5 ± 12.0	1.764	.166
	12 weeks	13	95.2 ± 12.7	12	91.3 ± 23.1	11	96.1 ± 15.5	17	92.6 ± 10.6	0.247	.863
CGI	Baseline	19	4.0 ± 0.8	14	3.6 ± 0.8	16	3.9 ± 0.7	19	3.7 ± 0.8		
	2 weeks	18	3.9 ± 0.6	13	3.6 ± 0.7	12	4.1 ± 0.5	19	3.6 ± 0.6	1.993	.125
	4 weeks	18	3.9 ± 0.8	12	3.7 ± 0.5	11	3.9 ± 0.5	19	3.6 ± 0.6	1.011	.395
	8 weeks	14	3.7 ± 0.5	12	3.6 ± 0.5	11	3.8 ± 0.6	17	3.6 ± 0.7	0.449	.719
	12 weeks	13	3.6 ± 0.5	12	3.4 ± 0.7	11	3.4 ± 0.5	17	3.8 ± 0.6	1.488	.229
SOFAS	Baseline	19	54.7 ± 9.1	14	58.6 ± 10.9	16	53.1 ± 11.9	19	54.7 ± 9.6		
	2 weeks	18	57.2 ± 11.8	13	58.5 ± 11.4	12	54.2 ± 5.2	19	57.9 ± 11.3	0.413	.744
	4 weeks	18	57.2 ± 10.2	12	54.2 ± 5.2	11	53.6 ± 6.7	19	56.3 ± 9.6	0.557	.645
	8 weeks	14	60.7 ± 9.2	12	57.5 ± 7.5	11	58.2 ± 7.5	17	59.4 ± 6.6	0.437	.727
	12 weeks	13	60.0 ± 10.0	12	59.2 ± 14.4	11	60.9 ± 9.4	17	63.5 ± 7.1	0.509	.678
CDSS	Baseline	19	14.8 ± 3.7	14	11.9 ± 3.9	16	12.3 ± 5.3	19	14.2 ± 3.5		
	2 weeks	18	13.9 ± 4.1	13	12.6 ± 3.3	12	13.7 ± 3.8	19	12.1 ± 3.9	0.850	.472
	4 weeks	18	13.7 ± 3.3	12	13.4 ± 3.2	11	12.9 ± 2.2	19	13.3 ± 2.2	0.177	.912
	8 weeks	14	13.1 ± 2.9	12	12.6 ± 3.3	11	14.2 ± 2.7	17	12.7 ± 3.2	0.661	.580
	12 weeks	13	12.6 ± 2.1	12	13.0 ± 3.4	11	11.8 ± 2.7	17	11.9 ± 2.0	0.651	.586
Median [IQR]											
Side effects	2 weeks	18	0 [0, 2]	13	0 [0, 2]	12	0 [0, 0]	18	0 [0, 0]	1.636	.384
	4 weeks	18	2 [0, 3]	12	0 [0, 2]	11	0 [0, 2]	18	0 [0, 1]	2.170	.110
	8 weeks	14	0 [0, 1]	12	0 [0, 2]	11	2 [0, 3]	16	0 [0, 2]	3.516	.377
	12 weeks	13	0 [0, 2]	12	0 [0, 0]	11	0 [0, 0]	17	0 [0, 0]	0.413	.976

**Table 3.** NaB Main Effects on PANSS Scores, CGI, SOFAS, and CDSS

Outcome	Time Point	No NaB [*N* = 33]		NaB [*N* = 35]		Group Difference	
		*n*	Mean ± *SD*	*n*	Mean ± *SD*	MD^a^ (95% CI)	*P-*value
PANSS positive	Baseline	33	23.2 ± 4.6	35	22.2 ± 4.4		
	2 weeks	31	21.8 ± 4.2	31	23.0 ± 4.9	−1.2 (−3.6, 1.1)	.285
	4 weeks	30	20.5 ± 3.5	30	23.0 ± 4.1	−2.5 (−4.5, −0.6)	**.012**
	8 weeks	26	21.5 ± 4.5	28	22.7 ± 4.1	−1.2 (−3.6, 1.1)	.303
	12 weeks	25	21.2 ± 3.9	28	21.0 ± 4.2	0.2 (−2.1, 2.4)	.883
PANSS negative	Baseline	33	23.7 ± 4.8	35	22.7 ± 5.3		
	2 weeks	31	23.0 ± 5.1	31	23.9 ± 4.1	−0.9 (−3.3, 1.4)	.426
	4 weeks	30	24.4 ± 4.4	30	24.0 ± 3.3	0.4 (−1.6, 2.4)	.691
	8 weeks	26	24.3 ± 4.0	28	24.4 ± 3.9	−0.1 (−2.2, 2.2)	.992
	12 weeks	25	22.7 ± 4.7	28	23.3 ± 3.4	−0.6 (−2.8, 1.6)	.569
PANSS general	Baseline	33	49.8 ± 6.2	35	51.0 ± 8.9		
	2 weeks	31	50.1 ± 9.4	31	53.3 ± 7.5	−3.2 (−7.5, 1.2)	.148
	4 weeks	30	51.3 ± 7.8	30	53.0 ± 7.9	−1.7 (−5.8, 2.4)	.407
	8 weeks	26	53.4 ± 7.1	28	52.6 ± 7.7	0.8 (−3.2, 4.9)	.677
	12 weeks	25	49.5 ± 10.7	28	49.6 ± 7.3	−0.1 (−5.1, 4.9)	.960
PANSS total	Baseline	33	96.7 ± 11.4	35	95.8 ± 16.7		
	2 weeks	31	94.9 ± 16.3	31	100.2 ± 13.9	−5.3 (−13.1, 2.4)	.170
	4 weeks	30	96.1 ± 12.0	30	99.9 ± 13.3	−3.8 (−10.4, 2.7)	.245
	8 weeks	26	99.3 ± 12.7	28	99.6 ± 12.3	−0.3 (−7.2, 6.5)	.913
	12 weeks	25	93.4 ± 18.2	28	94.0 ± 12.6	−0.6 (−9.1, 7.9)	.888
CGI	Baseline	33	3.8 ± 0.8	35	3.8 ± 0.8		
	2nd week	31	3.8 ± 0.6	31	3.8 ± 0.6	0.0 (−0.3, 0.3)	.836
	4th week	30	3.8 ± 0.7	30	3.7 ± 0.6	0.1 (−0.2, 0.4)	.550
	8th week	26	3.7 ± 0.5	28	3.8 ± 0.7	−0.1 (−0.4, 0.3)	.878
	12th week	25	3.5 ± 0.6	28	3.6 ± 0.6	−0.1 (−0.4, 0.2)	.585
SOFAS	Baseline	33	56.3 ± 9.9	35	54.0 ± 10.6		
	2nd week	31	57.7 ± 11.5	31	56.5 ± 9.5	1.2 (−4.1, 6.6)	.631
	4th week	30	56.0 ± 8.6	30	55.3 ± 8.6	0.7 (−3.8, 5.1)	.764
	8th week	26	59.2 ± 8.5	28	58.9 ± 6.9	0.3 (−3.9, 4.5)	.885
	12th week	25	59.6 ± 12.1	28	62.5 ± 7.9	−2.9 (−8.5, 2.7,)	.302
CDSS	Baseline	33	13.6 ± 4.0	35	13.3 ± 4.5		
	2 weeks	31	13.4 ± 3.7	31	12.7 ± 3.8	0.7 (−1.3, 2.6)	.506
	4 weeks	30	13.6 ± 3.2	30	13.1 ± 2.2	0.5 (−0.9, 1.9)	.544
	8 weeks	26	12.8 ± 3.1	28	13.3 ± 3.1	−0.4 (−2.1, 1.2)	.600
	12 weeks	25	12.8 ± 2.8	28	11.9 ± 2.3	0.9 (−0.4, 2.3)	.177

^a^Mean differences.

A similar set of analyses was performed to examine the effects of NAC upon the secondary outcomes. The NAC/No NAC results are shown in table 4. Again, there were no statistically significant differences between groups for the majority of outcomes at the majority of timepoints. However, significant differences between groups was observed for CGI score at 2 weeks with lower scores in the NAC group than in the no NAC group. A further significant difference was observed for the oral fluency (category) outcome at 12 weeks, where scores were significantly lower in the NAC group than in the no NAC group. There was a mean difference of 1.3 units between groups (supplementary table 3).

**Table 4.** NAC Main Effects on PANSS Scores, CGI, SOFAS, and CDSS

Outcome	Time Point	No NAC [*N* = 35]		NAC [*N* = 33]		Group Difference	
		*n*	Mean ± *SD*	*n*	Mean ± *SD*	MD^a^ (95% CI)	*P*-value
PANSS positive	Baseline	35	22.5 ± 4.3	33	22.9 ± 4.8		
	2 weeks	30	23.2 ± 2.8	32	21.6 ± 5.7	1.6 (−0.7, 3.9)	.163
	4 weeks	29	21.8 ± 2.8	31	21.7 ± 4.9	0.1 (−1.9, 2.1)	.962
	8 weeks	25	22.3 ± 3.2	29	22.0 ± 5.1	0.3 (−2.0, 2.7)	.765
	12 weeks	24	21.3 ± 3.4	29	21.0 ± 4.5	0.3 (−2.0, 2.5)	.772
PANSS negative	Baseline	35	23.0 ± 5.3	33	23.3 ± 4.9		
	2 weeks	30	23.6 ± 5.1	32	23.3 ± 4.2	0.3 (−1.9, 2.7)	.745
	4 weeks	29	24.3 ± 4.1	31	24.0 ± 3.7	0.3 (−1.7, 2.2)	.782
	8 weeks	25	25.2 ± 4.9	29	23.7 ± 2.8	1.5 (−0.7, 3.8)	.183
	12 weeks	24	23.8 ± 4.1	29	22.4 ± 4.0	1.4 (−0.9, 3.6)	.235
PANSS general	Baseline	35	50.3 ± 7.4	33	50.6 ± 8.1		
	2 weeks	30	51.9 ± 8.4	32	51.6 ± 8.8	0.3 (−4.1, 4.7)	.890
	4 weeks	29	52.8 ± 7.6	31	51.5 ± 8.2	1.3 (−2.7, 5.5)	.503
	8 weeks	25	55.3 ± 7.1	29	51.0 ± 7.2	4.3 (0.4, 8.2)	.033
	12 weeks	24	50.6 ± 8.1	29	48.7 ± 9.7	1.9 (−3.1, 6.9)	.450
PANSS total	Baseline	35	95.7 ± 13.9	33	96.8 ± 14.9		
	2 weeks	30	98.7 ± 13.9	32	96.4 ± 16.7	2.3 (−5.5, 10.1)	.559
	4 weeks	29	98.9 ± 11.6	30	97.2 ± 13.7	1.7 (−4.9, 8.3)	.608
	8 weeks	25	102.8 ± 11.4	28	96.6 ± 12.7	6.2 (−0.5, 12.8)	.069
	12 weeks	24	95.6 ± 13.7	28	92.1 ± 16.6	3.5 (−4.9, 12.1)	.405
CGI	Baseline	35	3.9 ± 0.7	33	3.7 ± 0.8		
	2 weeks	30	3.9 ± 0.6	32	3.6 ± 0.6	0.3 (0.1, 0.6)	.025
	4 weeks	29	3.9 ± 0.7	31	3.7 ± 0.6	0.2 (−0.1, 0.6)	.084
	8 weeks	25	3.8 ± 0.5	29	3.6 ± 0.6	0.2 (−0.1, 0.5)	.279
	12 weeks	24	3.5 ± 0.5	29	3.6 ± 0.6	−0.1 (−0.4, 0.2)	.450
SOFAS	Baseline	35	54.0 ± 10.3	33	56.4 ± 10.3		
	2 weeks	30	56.0 ± 9.7	32	58.1 ± 11.2	−2.1 (−7.5, 3.2)	.429
	4 weeks	29	55.9 ± 9.1	31	55.5 ± 8.1	0.4 (−4.1, 4.8)	.865
	8 weeks	25	59.6 ± 8.4	29	58.6 ± 6.9	1.0 (−3.2, 5.2)	.641
	12 weeks	24	60.4 ± 9.6	29	61.7 ± 10.7	−1.3 (−6.9, 4.4)	.644
CDSS	Baseline	35	13.7 ± 4.6	33	13.2 ± 3.8		
	2 weeks	30	13.8 ± 3.9	32	12.3 ± 3.6	1.5 (−0.4, 3.4)	.122
	4 weeks	29	13.3 ± 2.9	31	13.3 ± 2.6	0.1 (−1.4, 1.5)	.937
	8 weeks	25	13.6 ± 2.8	29	12.7 ± 3.2	0.9 (−0.8, 2.6)	.280
	12 weeks	24	12.3 ± 2.4	29	12.3 ± 2.7	0.0 (−1.5, 1.3)	.893

^a^Mean difference.

## Discussion

This study demonstrated the feasibility of recruiting and retaining individuals with early psychosis in an interventional study examining novel pharmacotherapeutics aimed at minimizing illness severity, improving functioning, and cognition. A high proportion of eligible individuals agreed to participate in the study and subsequently be randomized into 1 of the 4 arms of the clinical trial. Seventy-seven (65%) of those eligible to participate attended for baseline screening and 68 (88%) of those screened were willing to be randomized, demonstrating that recruitment to a full trial is possible and likely to be completed successfully. There were no challenges in engaging the target population and no changes to recruitment procedures were required, which speaks to the success of engaging people with early psychosis in the study. We also aimed to assess whether established measures of clinical and functional outcome were appropriate measures in this context. We encountered no difficulties in participant comprehension of assessments nor challenges in implementing our assessment schedule, indicating that participants were not dissatisfied with these procedures and researchers found implementation unproblematic. In terms of acceptability, we are encouraged by low attrition and relatively high adherence rates, which infers a high degree of acceptability of the interventions. There were no significant differences in side effects between the intervention arms and no discontinuation due to intolerable side effects. With the data presented here we cannot draw firm conclusions about the clinical efficacy of either agent, and a larger appropriately powered clinically efficacy trial would be needed to determine this.

Although prior studies have demonstrated that NaB as an adjunct to standard care in schizophrenia can improve symptoms, the findings have been inconsistent. Findings have largely come from the same research group in Taiwan. The first of these RCTs recruited 52 individuals with chronic schizophrenia who were stable on antipsychotic medication and administered 6 weeks of treatment with NaB 1000 mg daily.^9^ Individuals in the experimental arms showed improvement in PANSS total and subscale scores with large effect sizes (range, 1.16–1.69).^9^ Encouragingly there were also improvements noted in negative symptoms, functional domains and neurocognitive assessments which sparked a great deal of interest in examining the role of NaB as a treatment for these disabling symptom domains.^9^ The same investigators led a 12-week RCT (*n* = 63) to examine the efficacy of NaB in combination with sarcosine in improving cognitive and global functioning in individuals with chronic schizophrenia.^48^ Sarcosine plus NaB but not sarcosine alone, improved cognitive and global functioning.^48^ The findings from this study are not directly comparable to the present as there was not an NaB only arm. The most recent study was a 6-week RCT of NaB in addition to clozapine (*n* = 60) in individuals with treatment-resistant schizophrenia and demonstrated that 1000 and 2000 mg/day of NaB improved negative symptoms of schizophrenia, and the 2000 mg/day also improved PANSS-total score, PANSS-positive score, and quality of life.^20^ All of the above studies were limited by small sample sizes and short durations of follow-up. In the most recent study, NaB improved negative symptoms as measured by SANS and our study employed the PANSS negative subscale to rate negative symptoms, which may have contributed to the differing results. Furthermore, only 2000 mg of NaB improved PANSS-total subscale scores, PANSS-positive subscale scores, and quality of life, while in the present study only 1000 mg of NaB was administered. The key factors to consider when interpreting the results of the present study in the context of the existing literature are the doses administered, the clinical instruments used to assess symptoms severity and cognition, and recruitment of participants at a later stage of illness as well as those treated with clozapine albeit at a subtherapeutic dose. The once daily dosing schedule employed in the present study should be considered as well. Prior studies have utilized a twice daily dosing, which may be more appropriate for a compound with the pharmacokinetics of NaB.^49^ Recent studies have shown that NaB is rapidly metabolized showing peak plasma levels at 0.5 h and having a plasma half-life of 0.3 h.^49^ More recently, a larger 12-week RCT of NaB added to standard care (*n* = 100) in individuals with early psychosis found no evidence that NaB improved positive symptoms, negative symptoms, functioning, or quality life.^50^ Like our study, it is worth noting that this study was not powered to detect a small effect size and the authors rightfully commented that improvements in negative symptoms and functioning may have emerged with continued treatment, beyond the duration of the trial.

NAC remains a pharmacotherapeutic compound of interest across psychiatric disorders. A 2020 meta-analysis of RCTs investigating NAC in schizophrenia included data from 7 clinical trials (NAC, *n* = 220; placebo, *n* = 220).^34^ The meta-analysis demonstrated that PANSS negative and total scores were significantly reduced in individuals receiving NAC after 24 weeks of treatment.^34^ They also concluded that neurocognition, specifically working memory, improved following NAC treatment.^34^ Since the publication of this meta-analysis, a small study from Russia demonstrated a significant improvement PANSS positive (*P* = .013), negative (*P* = .002), general psychopathology (*P* = .004), and working memory (*P* = .037).^51^ In the most recent published study, NAC added to clozapine in treatment-refractory individuals with chronic schizophrenia (*n* = 85) did not significantly improve negative symptoms (*P* = .62), cognitive functioning (*P* = .71) or quality of life over a 1-year period of treatment.^52^ In the early psychosis population, a 52-week RCT (*N* = 60) of NAC reported significantly improved PANSS total and negative symptom scores in the treatment arm, however, there was no effect on positive symptoms nor cognitive functioning.^53^ The effect of NAC on negative symptoms and neurocognition in early psychosis was also examined in a 6-month RCT (*n* = 63) where NAC was associated with significant improvements in neurocognition (processing speed) although no changes in positive symptoms, negative symptoms, or functioning were found.^54^ Similar to the studies of NaB described above, many of the NAC trials discussed were limited by small sample sizes, which makes drawing definitive conclusions about clinical efficacy challenging. Prior studies of NAC have administered doses ranging from 600 to 3600 mg/day with treating extending to 1 year. Breier et al.^53^ evaluated NAC at 3600 mg/day in a 52 weeks, double-blind, placebo-controlled trial in early phase schizophrenia spectrum disorders (*N* = 60). NAC significantly improved PANSS total, negative and disorganized thought symptom scores.^53^ Future studies may consider higher doses and longer treatment duration than those used in the current study.

One of the main strengths of the present RCT is that it is the first to examine the combined and comparative effects of NaB and NAC in early psychosis. We used gold-standard structured clinical assessments to confirm diagnosis of early schizophrenia in participants and established clinical outcome measures. There are several limitations to the study that warrant consideration. When interpreting these results, it should be noted that the trial was not powered to show a statistical difference between groups. Future studies that are appropriately powered may consider higher doses of NAC, twice daily administration of NaB given its short half-life, and a longer treatment duration than what was used in the current study. Estimates of adherence were based on counting capsules returned and rely on the assumption that the medications returned are the only ones not taken. The sample recruited to this study were markedly-severely ill and the scores remained in this range despite 12 weeks of treatment with antipsychotic medication, potentially indicating poor treatment adherence. More robust measures of treatment adherence should be employed in future trials. Poor adherence to study medications may have contributed to the discrepant findings between the current study and previously published literature.

Finding new and effective treatments for early schizophrenia is crucial, given the morbidity, mortality, and disability associated with this severe and enduring condition. Despite adherence to psychotropic medication, it is estimated that a third of patients with psychotic disorders continue to experience residual symptoms.^55^ Effective intervention in early psychosis is a critical juncture where pharmacological, social, and cognitive therapies can have a significant effect on longer term outcomes.^1,31,55^ NAC and NaB are thought to be promising compounds that may have therapeutic effect in early psychosis. To our knowledge this is the first trial to explore the combination of these agents in the treatment of early schizophrenia. The findings support the feasibility of conducting a large-scale multicenter clinical efficacy trial. Although there were no numerical and statistical differences between the treatment groups, and a large-scale trial with longer duration of follow-up may be needed to examine if significant differences between treatment groups emerge.

## Supplementary Material

sgae004_suppl_Supplementary_Tables_1-4

## Data Availability

Available on reasonable request from the corresponding author.

## Dissemination

The results of this trial will be presented at relevant academic and nonacademic meetings in partnership with service users and published in open-access peer-reviewed journals and for lay public.
