# Supplementary material for: Add-on Sodium Benzoate and N-Acetylcysteine in Patients With Early Schizophrenia Spectrum Disorder: A Multicenter, Double-Blind, Randomized Placebo-Controlled Feasibility Trial
Source: Schizophr Bull Open. 2024 Feb 9;5(1):sgae004. doi: 10.1093/schizbullopen/sgae004 (PMC11207662; doi:10.1093/schizbullopen/sgae004)
Supplement: sgae004_suppl_Supplementary_Tables_1-4 [file sgae004_suppl_Supplementary_Tables_1-4.docx]

*Supplementary Table 1: Total number of side effects reported by group*

| **Number of side effects per treatment group** | **Placebo**  **(n = 63)** | **NAC only**  **(n = 49)** | **NaB only**  **(n = 45)** | **NAC + NaB**  **(n = 69)** | ***P-value*** |
| --- | --- | --- | --- | --- | --- |
|  | n (%) | | | |  |
| Headache | 16 (25.4%) | 06 (12.2%) | 11 (24.4%) | 08 (11.6%) | .087 |
| Agitation, Restlessness and Anxiety | 11 (17.5%) | 04 (8.2%) | 04 (8.9%) | 05 (7.2%) | .223 |
| Irritability | 09 (14.3%) | 10 (20.4%) | 06 (13.3%) | 08 (11.6%) | .596 |
| Hyperventilation | 00 (0.0%) | 00 (0.0%) | 00 (0.0%) | 01 (1.4%) | .515 |
| Shortness of breath | 03 (4.8%) | 00 (0.0%) | 00 (0.0%) | 02 (2.9%) | .244 |
| Visual disturbance | 00 (0.0%) | 01 (2.0%) | 01 (2.2%) | 01 (1.4%) | .724 |
| Nerve Senstivity | 00 (0.0%) | 00 (0.0%) | 00 (0.0%) | 01 (1.4%) | .515 |
| Tingling in hands and feet | 01 (1.6%) | 00 (0.0%) | 01 (2.2%) | 01 (1.5%) | .808 |
| Ringing in the ears | 03 (4.8%) | 02 (4.1%) | 02 (4.4%) | 01 (1.4%) | .727 |
| Trouble sleeping | 04 (6.3%) | 01 (2.0%) | 03 (6.7%) | 04 (5.8%) | .712 |
| Tremor | 02 (3.2%) | 00 (0.0%) | 01 (2.2%) | 01 (1.5%) | .642 |
| Urinary frequency | 01 (1.6%) | 00 (0.0%) | 01 (2.2%) | 01 (1.4%) | .807 |
| Tachycardia | 00 (0.0%) | 03 (6.1%) | 04 (8.9%) | 02 (2.9%) | .102 |
| Nausea or Vomitting | 05 (7.9%) | 07 (14.3%) | 03 (6.7%) | 02 (2.9%) | .144 |
| Rash | 01 (1.6%) | 00 (0.0%) | 00 (0.0%) | 00 (0.0%) | .458 |
| Low Blood pressure | 01 (1.6%) | 01 (2.0%) | 00 (0.0%) | 00 (0.0%) | .548 |

*Supplememtary Table 2: NaB main effects cognitive parameters*

| **Outcome** | **Timepoint** | **No NaB [N=33]** | | **NaB [N=35]** | | **Group Difference** | |
| --- | --- | --- | --- | --- | --- | --- | --- |
|  |  | **n** | **Mean ± SD** | **n** | **Mean ± SD** | **MD ^(*)^ (95% CI)** | ***P-value*** |
| SCWT Color error | Baseline | 20 | 5.0 ± 2.1 | 24 | 4.1 ± 2.4 |  |  |
|  | 12 weeks | 16 | 3.7 ± 2.5 | 22 | 3.6 ± 2.7 | 0.1 (-1.6, 2.0) | .911 |
|  |  |  |  |  |  |  |  |
| SCWT Word error | Baseline | 20 | 1.4 ± 1.3 | 24 | 1.2 ± 1.6 |  |  |
|  | 12 weeks | 16 | 0.8 ± 1.3 | 22 | 0.8 ± 1.3 | 0.0 (-0.9, 0.8) | .871 |
|  |  |  |  |  |  |  |  |
| Error difference | Baseline | 20 | 3.6 ± 1.9 | 24 | 2.8 ± 2.1 |  |  |
|  | 12 weeks | 22 | 2.8 ± 2.1 | 22 | 2.6 ± 2.5 | 0.2 (-1.3, 1.8) | .773 |
|  |  |  |  |  |  |  |  |
| Color time | Baseline | 20 | 163.8 ± 33.1 | 24 | 154.0 ± 32.4 |  |  |
|  | 12 weeks | 16 | 148.8 ± 21.9 | 22 | 143.4 ± 49.1 | 5.4 (-21.3, 32.1) | .684 |
|  |  |  |  |  |  |  |  |
| Word time | Baseline | 20 | 111.0 ± 22.3 | 24 | 105.2 ± 30.9 |  |  |
|  | 12 weeks | 16 | 104.7 ± 20.6 | 22 | 95.4 ± 35.7 | 9.3 (-10.9, 29.6) | .356 |
|  |  |  |  |  |  |  |  |
| Time difference | Baseline | 20 | 52.9 ± 22.4 | 24 | 49.5 ± 15.9 |  |  |
|  | 12 weeks | 16 | 42.9 ± 10.0 | 22 | 47.9 ± 19.9 | -5.0 (-16.0, 6.0) | .364 |
|  |  |  |  |  |  |  |  |
| Block design | Baseline | 30 | 0.4 ± 0.9 | 31 | 0.7 ± 1.1 |  |  |
|  | 12 weeks | 24 | 0.7 ± 1.2 | 28 | 1.1 ± 1.5 | -0.4 (-1.2, 0.3) | .242 |
|  |  |  |  |  |  |  |  |
| Coughlan Verbal | Baseline | 30 | 32.8 ± 9.6 | 31 | 33.7 ± 9.1 |  |  |
|  | 12 weeks | 24 | 31.0 ± 6.8 | 28 | 33.7 ± 6.4 | -2.6 (-6.4, 1.0) | .152 |
|  |  |  |  |  |  |  |  |
| Coughlan visual | Baseline | 30 | 14.1 ± 7.4 | 31 | 17.6 ± 14.5 |  |  |
|  | 12 weeks | 24 | 13.8 ± 7.1 | 28 | 15.5 ± 9.9 | -1.7 (-6.6, 3.1) | .464 |
|  |  |  |  |  |  |  |  |
| Oral fluen - Words | Baseline | 30 | 3.8 ± 4.0 | 31 | 4.7 ± 5.3 |  |  |
|  | 12 weeks | 24 | 3.2 ± 3.5 | 28 | 4.6 ± 4.6 | -1.4 (-3.7, 0.8) | .193 |
|  |  |  |  |  |  |  |  |
| Oral fluen -Category | Baseline | 30 | 11.6 ± 4.2 | 30 | 11.8 ± 5.2 |  |  |
|  | 12 weeks | 24 | 10.5 ± 2.3 | 28 | 10.7 ± 2.1 | -0.2 (-1.5, 1.0) | .723 |

*(*) Mean differences calculated as outcome in NaB group minus outcome in No NaB group*

*Stroop Color and Word Test (SCWT)*

*Supplememtary Table 3: NAC effects – Cognitive parameters*

| **Outcome** | **Timepoint** | **No NAC [N=35]** | | **NAC [N=33]** | | **Group Difference** | |
| --- | --- | --- | --- | --- | --- | --- | --- |
|  |  | **n** | **Mean ± SD** | **n** | **Mean ± SD** | **MD (*) (95% CI)** | ***P-value*** |
| SCWT Color error | Baseline | 20 | 4.4 ± 2.2 | 24 | 4.6 ± 2.4 |  |  |
|  | 12 weeks | 16 | 3.2 ± 2.6 | 22 | 4.0 ± 2.6 | -0.8 (-2.5, 0.9) | .371 |
|  |  |  |  |  |  |  |  |
| SCWT Word error | Baseline | 20 | 1.3 ± 1.5 | 24 | 1.3 ± 1.5 |  |  |
|  | 12 weeks | 16 | 0.7 ± 1.3 | 22 | 0.9 ± 1.3 | -0.2 (-1.0, 0.7) | .675 |
|  |  |  |  |  |  |  |  |
| Error difference | Baseline | 20 | 3.1 ± 1.9 | 24 | 3.3 ± 2.1 |  |  |
|  | 12 weeks | 22 | 2.4 ± 2.4 | 22 | 2.9 ± 2.3 | -0.5 (-2.1, 1.0) | .486 |
|  |  |  |  |  |  |  |  |
| Color time | Baseline | 20 | 155 ± 33 | 24 | 162 ± 33 |  |  |
|  | 12 weeks | 16 | 146.3 ± 49.7 | 22 | 145.2 ± 31.7 | 1.1 (-25.7, 27.8) | .935 |
|  |  |  |  |  |  |  |  |
| Word time | Baseline | 20 | 104 ± 24 | 24 | 111 ± 30 |  |  |
|  | 12 weeks | 16 | 98.9 ± 33.1 | 22 | 99.6 ± 28.9 | -0.6 (-21.1, 19.9) | .952 |
|  |  |  |  |  |  |  |  |
| Time difference | Baseline | 20 | 51 ± 23 | 24 | 51 ± 16 |  |  |
|  | 12 weeks | 16 | 46.5 ± 21.6 | 22 | 45.2 ± 12.1 | 1.3 (-9.9, 12.4) | .818 |
|  |  |  |  |  |  |  |  |
| Block design | Baseline | 31 | 0.1 ± 0.5 | 30 | 1.0 ± 1.2 |  |  |
|  | 12 weeks | 24 | 0.5 ± 1.1 | 28 | 1.3 ± 1.5 | -0.8 (-1.5, -0.1)) | **.042** |
|  |  |  |  |  |  |  |  |
| Coughlan Verbal | Baseline | 31 | 31.3 ± 8.8 | 30 | 35.3 ± 9.4 |  |  |
|  | 12 weeks | 24 | 33.3 ± 5.8 | 28 | 31.8 ± 7.3 | 1.5 (-2.2, 5.2) | .813 |
|  |  |  |  |  |  |  |  |
| Coughlan visual | Baseline | 31 | 15.5 ± 11.6 | 30 | 16.4 ± 11.8 |  |  |
|  | 12 weeks | 24 | 15.1 ± 9.4 | 28 | 14.4 ± 8.2 | 0.7 (-4.1, 5.7) | .753 |
|  |  |  |  |  |  |  |  |
| Oral fluen - Words | Baseline | 31 | 3.8 ± 4.6 | 30 | 4.7 ± 4.9 |  |  |
|  | 12 weeks | 24 | 3.9 ± 4.4 | 28 | 4.0 ± 3.9 | -0.1 (-2.5, 2.2) | .890 |
|  |  |  |  |  |  |  |  |
| Oral fluen - Category | Baseline | 31 | 11.1 ± 4.4 | 30 | 12.3 ± 5.0 |  |  |
|  | 12 weeks | 24 | 11.3 ± 2.2 | 28 | 10.0 ± 2.1 | 1.3 (0.1, 2.4) | **.040** |

*(*) Calculated as outcome in NaC group minus outcome in No NaC group*

*Stroop Color and Word Test (SCWT)*

*Supplememtary Table 4: Cognitive parameters by group*

| **Outcome** | **Timepoint** | **NAC + NaB**  **(N = 19)** | | **Placebo**  **(N = 19)** | | **Placebo + NAC**  **(N = 14)** | | **Placebo + NaB**  **(N = 16)** | | ***F*** | ***P*-value** |
| --- | --- | --- | --- | --- | --- | --- | --- | --- | --- | --- | --- |
|  |  | **n** | **Mean ± SD** | **n** | **Mean ± SD** | **n** | **Mean ± SD** | **n** | **Mean ± SD** |  |  |
| SCWT Color error | Baseline | 15 | 4.7 ± 2.5 | 11 | 5.5 ± 1.8 | 09 | 4.3 ± 2.5 | 09 | 3.1 ± 2.0 | 1.892 | .147 |
|  | 12 weeks | 14 | 3.6 ± 2.7 | 08 | 2.75 ± 2.5 | 08 | 4.6 ± 2.3 | 08 | 3.6 ± 2.8 | .696 | .561 |
|  |  |  |  |  |  |  |  |  |  |  |  |
| Color time | Baseline | 15 | 160.9 ± 35.2 | 11 | 164.6 ± 36.7 | 09 | 162.7 ± 30.1 | 09 | 142.6 ± 24.9 | .912 | .444 |
|  | 12 weeks | 14 | 143.9 ± 36.1 | 08 | 150.1 ± 20.9 | 08 | 147.5 ± 24.2 | 08 | 142.5 ± 69.4 | .061 | .980 |
|  |  |  |  |  |  |  |  |  |  |  |  |
| SCWT Word error | Baseline | 15 | 1.4 ± 1.7 | 11 | 1.6 ± 1.4 | 09 | 1.0 ± 1.2 | 09 | 0.9 ± 1.5 | .541 | .657 |
|  | 12 weeks | 14 | 0.7 ± 1.1 | 08 | 0.4 ± 0.7 | 08 | 1.1 ± 1.6 | 08 | 1.0 ± 1.6 | .552 | .650 |
|  |  |  |  |  |  |  |  |  |  |  |  |
| Word time | Baseline | 15 | 109.7 ± 32.4 | 11 | 109.6 ± 32.4 | 09 | 112.6 ± 26.5 | 09 | 97.8 ± 28.6 | .519 | .671 |
|  | 12 weeks | 14 | 96.8 ± 32.1 | 08 | 105.0 ± 18.9 | 08 | 104.4 ± 23.5 | 08 | 92.9 ± 43.7 | .303 | .823 |
|  |  |  |  |  |  |  |  |  |  |  |  |
| Error difference | Baseline | 15 | 3.2 ± 2.2 | 11 | 3.8 ± 1.9 | 09 | 3.3 ± 2.0 | 09 | 2.2 ± 1.7 | 1.082 | .368 |
|  | 12 weeks | 14 | 2.7 ± 2.6 | 08 | 2.4 ± 2.6 | 08 | 3.3 ± 1.6 | 08 | 2.4 ± 2.3 | .245 | .865 |
|  |  |  |  |  |  |  |  |  |  |  |  |
| Time difference | Baseline | 15 | 51.3 ± 16.9 | 11 | 55.1 ± 28.1 | 09 | 50.1 ± 13.6 | 09 | 46.4 ± 14.4 | .335 | .800 |
|  | 12 weeks | 14 | 47.1 ± 13.3 | 08 | 43.9 ± 10.9 | 08 | 41.9 ± 9.6 | 08 | 49.1 ± 29.3 | .308 | .819 |
|  |  |  |  |  |  |  |  |  |  |  |  |
| Block design | Baseline | 18 | 1.2 ± 1.3 | 18 | 0.2 ± 0.6 | 12 | 0.8 ± 1.1 | 13 | 0.0 ± 0.0 | 5.025 | .004 |
|  | 12 weeks | 17 | 1.6 ± 1.6 | 13 | 0.6 ± 1.2 | 11 | 0.8 ± 1.2 | 11 | 0.5 ± 1.0 | 2.211 | .099 |
|  |  |  |  |  |  |  |  |  |  |  |  |
| Coughlan Verbal | Baseline | 18 | 34.8 ± 9.6 | 18 | 30.6 ± 9.2 | 12 | 36.2 ± 9.6 | 13 | 32.3 ± 8.4 | 1.122 | .348 |
|  | 12 weeks | 17 | 32.8 ± 6.7 | 13 | 31.8 ± 5.6 | 11 | 30.2 ± 8.2 | 11 | 35.1 ± 5.8 | 1.076 | .368 |
|  |  |  |  |  |  |  |  |  |  |  |  |
| Coughlan visual | Baseline | 18 | 20.0 ± 13.7 | 18 | 16.2 ± 8.2 | 12 | 11.0 ± 4.7 | 13 | 14.4 ± 15.4 | 1.584 | .203 |
|  | 12 weeks | 17 | 13.9 ± 8.7 | 13 | 12.6 ± 6.7 | 11 | 15.1 ± 7.6 | 11 | 18.1 ± 11.4 | .864 | .466 |
|  |  |  |  |  |  |  |  |  |  |  |  |
| Oral fluen - Words | Baseline | 18 | 4.6 ± 5.4 | 18 | 3.0 ± 3.7 | 12 | 4.9 ± 4.3 | 13 | 4.9 ± 5.5 | .611 | .611 |
|  | 12 weeks | 17 | 4.1 ± 4.3 | 13 | 2.5 ± 3.5 | 11 | 4.0 ± 3.4 | 11 | 5.5 ± 4.9 | 1.122 | .349 |
|  |  |  |  |  |  |  |  |  |  |  |  |
| Oral fluen -Category | Baseline | 18 | 11.7 ± 5.3 | 18 | 10.5 ± 3.7 | 12 | 13.3 ± 4.6 | 13 | 12.0 ± 5.2 | .887 | .453 |
|  | 12 weeks | 17 | 10.0 ± 1.8 | 13 | 10.9 ± 2.2 | 11 | 10.0 ± 2.5 | 11 | 11.7 ± 2.2 | 1.790 | .162 |

*(*) Mean differences calculated as outcome in NaB+NaC, Plecebo, Placebo+NaC and Placeebo+NaB groups*

*Stroop Color and Word Test (SCWT)*
